# How Human H1 Histone Recognizes DNA

**DOI:** 10.3390/molecules25194556

**Published:** 2020-10-05

**Authors:** Olesya P. Luzhetskaya, Sergey E. Sedykh, Georgy A. Nevinsky

**Affiliations:** Institute of Chemical Biology and Fundamental Medicine, SD of Russian Academy of Sciences, 8 Lavrentiev Ave., 630090 Novosibirsk, Russia; lesy0102@gmail.com (O.P.L.); sirozha@gmail.com (S.E.S.)

**Keywords:** human H1 histone, oligonucleotides, patterns of DNA recognition

## Abstract

Linker H1 histone is one of the five main histone proteins (H1, H2A, H2B, H3, and H4), which are components of chromatin in eukaryotic cells. Here we have analyzed the patterns of DNA recognition by free H1 histone using a stepwise increase of the ligand complexity method; the affinity of H1 histone for various single- and double-stranded oligonucleotides (d(pN)_n_; *n* = 1–20) was evaluated using their competition with 12-mer [^32^P]labeled oligonucleotide and protein–oligonucleotide complex delaying on nitrocellulose membrane filters. It was shown that minimal ligands of H1 histone (like other DNA-dependent proteins and enzymes) are different mononucleotides (dNMPs; *K*_d_ = (1.30 ± 0.2) × 10^−2^ M). An increase in the length of single-stranded (ss) homo- and hetero-oligonucleotides (d(pA)_n_, d(pT)_n_, d(pC)_n_, and d(pN)_n_ with different bases) by one nucleotide link regardless of their bases, leads to a monotonic increase in their affinity by a factor of *f* = 3.0 ± 0.2. This factor *f* corresponds to the *K*_d_ value = 1/*f* characterizing the affinity of one nucleotide of different ss d(pN)_n_ for H1 at *n* = 2–6 (which are covered by this protein globule) is approximately 0.33 ± 0.02 M. The affinity of five out of six DNA nucleotide units is approximately 25 times lower than for one of the links. The affinity of duplexes of complementary homo- and hetero-d(pN)_20_ is only 1.3–3.3-fold higher in comparison with corresponding ss oligonucleotides. H1 histone forms mainly weak additive contacts with internucleoside phosphate groups of ssDNAs and one chain of double-stranded DNAs, but not with the bases.

## 1. Introduction 

In eukaryotic cells, genetic information encoded in DNA is packed as chromatin in the cell nuclei, the diameters of which do not exceed 10 μm [1,2,3,4]. The first stage of DNA compaction is interaction with histones, leading to the formation of nucleosomes, the structure of which, after their discovery in 1974, remained unclear for a long time. The exact crystal structures were determined after 1997 by X-ray diffractometry and have long been regarded as basic conformational states [1,2,3,4]. However, the variety of allowed crystal structures of nucleosomes has increased the intensity study in this area and led to the development of the concept of the nucleosome as a dynamic unit [5]. The scientific interest is due to the fact that many mechanisms of gene regulation are realized at the nucleosomal level. A well-known example is the "nucleosomal barrier"—a mechanism that prevents RNA polymerase from accessing DNA in the absence of specific conformational changes.

There are only five classes of histones H2A, H2B, H3, H4, and linker histones. The latter group includes H1 and H5, which is specific to particular organisms [1,2,3,4,5]. Core histones are proteins with a molecular weight of 10–15 kDa, while H1/H5 has a higher molecular weight (≈23 kDa). Core histones form a complex octamer; 146 base pair segment of DNA is wrapped around the histone octamer forming a chromatosome—a complex about 7 nm in diameter [6]. The localization of histone H1 can be judged because when treated with alkali or acid, H1 is the first protein to be displaced from chromatin [7,8,9]. 

H1 exists in multiple isoforms. In mammals, there are at least six somatic H1 subtypes: H1° and H1a–e, an oocyte-specific subtype, H1oo, and a male germ-line specific subtype, H1t [10,11,12,13]. These subtypes differ in small amino acid substitutions in the non-globular N- and C-terminal tails of the histone, their expression in time [14], level of phosphorylation [15], and turnover rate [16,17]. In vitro experiments support the hypothesis that these subtypes can differ in their ability to condense chromatin [18,19,20]. Gene expression and developmental studies also support that the different H1 subtypes play particular roles in chromatin structure [21,22,23,24].

H1 is very rich in lysine: it makes up more than 25% of the amino acid residues in its composition, and the lysine residues are concentrated near the C-terminus, which has a length of about 100 residues [25]. The charge of histone H1 is higher than that of histones H2A, H2B, H3, and H4. The central part of H1 contains many hydrophobic residues, as a result of which, it forms globules in solution; despite the presence of nonpolar residues, the globular domain is positively charged at physiological pH (pI > 10). It is assumed that the globular and nonpolar central region of the histone interacts with molecules of other histones, while the lysine-rich region is responsible for DNA binding [9,25].

A free exchange of H1 histone between chromatin and DNA binding sites was used to estimate the relative affinities of H1 somatic subtypes for purified chromatin fragments of 30–35 nucleosomes in physiological salt at constant H1 stoichiometry [26]. H1 is freely exchanged between chromatin and binding sites of SAR (fragment of 657 bp from the Drosophila histone cluster). The order of relative affinity for SAR DNA, was expressed relatively to affinity for H1a, the weakest binding subtype, was: H1d (20.0) ≈ H1° (20.0) > H1e (15.0) > H1b (5.6) > H1c (4.1) > H1a (1.0).

The authors of the review [27] described how the polyelectrolyte properties of chromatin and DNA may be illustrated by the experimental results of folding and self-association of a well-defined model chromatin in the form of recombinant arrays of nucleosomes, and how these properties can be understood from computer simulations. The compaction of chromatin was shown may have significant similarity to DNA condensation. However, the structure of condensed chromatin is sensitive to detailed molecular features of nucleosomal-nucleosome interactions, which include the influence of histone tails and their modifications [27].

Using a simple biophysical model, the effect of electrostatic binding of histone H1 proteins on the length of nucleosomal repeats in chromatin was described [28]. According to the received data, the length of the wrapped DNA may optimize its energy of binding to the histone nucleus and the elastic energy penalty of DNA wrapping. The magnitude of the model predicted effect [28] agrees with experimental data on linear repeat lengths changes in nucleosome as a function of the nucleosome/H1 ratio [29]. The data of [27,28,29] testify that the interaction of H1 histone with DNA is mostly electrostatic. However, these data do not provide detailed quantitative characteristics of DNA recognition by free histone H1 and it in nucleosomes. 

Currently, the most informative method for enzymes and proteins analysis is X-ray diffraction analysis [30,31,32,33]. It helps to obtain data on protein–nucleic acid interactions, but, however, this method is not suitable for quantitative assessment of the contribution of individual-specific and non-specific contacts to the affinity of nucleic acids for enzymes and proteins. To study enzymes, the substrate properties of oligonucleotides of different lengths and their analogs are usually used. The lack of substrate properties of an oligonucleotide can sometimes be explained by a possible lack of affinity for the enzyme. However, they may have even higher affinity than optimal substrates, but not undergo a specific protein-dependent transformation. The most informative method for studying DNA-dependent enzymes is the method of inhibitory analysis, a quantitative assessment of the most important factors that are important for DNA recognition by various enzymes and for understanding the physicochemical laws of protein–nucleic acid interactions [30,31,32,33]. There is little data on the quantitative assessment of the contributions of the kinetic (*k*_cat_) and thermodynamic (complexation) stages of the catalytic process to the affinity for DNA and the contribution of the DNA adaptation stage to the conformation optimal for the enzyme to the specificity of the action of enzymes [30,31,32,33]. 

It is possible to obtain a concrete interpretation of X-ray structural analysis pictures of contacts between proteins and DNA only with the help of detailed quantitative analysis. It has been shown that the principle of additivity of Gibbs free energies underlies the recognition of extended nucleic acids by enzymes and proteins, and the study of the mechanism of protein–nucleic acid interactions can be carried out at the molecular level by the method of a stepwise increase of the ligand complexity (SILC) (reviewed in [30,31,32,33]).

The SILC method was first used to describe different patterns of the interaction of DNA with replication [34,35,36,37], restriction [38], integration [39], topoisomerization [40,41], and different repair enzymes: uracil DNA glycosylase [42], Fpg protein from *E. coli* [43,44], human 8-oxoguanine-DNA glycosylase [45], human apurinic/apyrimidinic endonuclease [46], and RecA protein [47,48]. On the sum of the data obtained, the general patterns of DNA recognition by enzymes and subsequent catalysis were established.

It was shown that all these enzymes first form complexes with all specific and unspecific DNAs ([30,31,32,33,34,35,36,37,38,39,40,41,42,43,44,45,46,47,48], for review, see [30,31]). Depending on enzyme molecular weight, 7–20 nucleotide units of DNAs usually interact with their DNA-binding clefts providing high affinity (5–8 orders of magnitude) due to numerous weak additive non-specific interactions between each enzyme and various structural elements of DNA nucleotide units. The transition from unspecific to specific DNAs is usually accompanied by the strengthening of some contacts formed by unspecific DNA and by the formation of some new specific contacts [30,31,32,33,34,35,36,37,38,39,40,41,42,43,44,45,46,47,48]. All specific interactions between all enzymes and peculiar DNAs are usually weak; the contributions of specific interactions to the total affinity of DNAs for enzymes are rather small and do not exceed 1–2 orders of magnitude ([30,31,32,33,34,35,36,37,38,39,40,41,42,43,44,45,46,47,48], for review, see [30,31]). After the formation of the primary complex, all DNAs and enzymes undergo multiple conformational changes to reach the catalytically competent structure of their complexes. Finally, the reaction rates in the case of specific DNAs are accelerated by 6–8 orders of magnitude. The substrate specificities of all enzymes analyzed are provided due to the stages of the enzyme-dependent adjustment of DNAs and enzymes conformations and directly by chemical stages of the catalysis ([30,31,32,33,34,35,36,37,38,39,40,41,42,43,44,45,46,47,48], for review, see [30,31]).

However, in addition to canonical enzymes, many DNA-binding proteins have been described with no or very weak catalytic activity. The regularities of DNA complex formation with human milk lactoferrin [49] and lactalbumin [50], as well as human blood albumin [51] and IgGs against DNA [52], were analyzed.

It was shown that the recognition of DNAs by such proteins and antibodies occurs in accordance with the general patterns with those for enzymes analyzed. The interaction of all enzymes and proteins with single-stranded (ss) DNA is a superposition of weak non-specific electrostatic and hydrogen bonds, as well as hydrophobic and/or Van der Waals interactions with individual structural elements and is described by a single decreasing geometric progression ([30,31,32,33,34,35,36,37,38,39,40,41,42,43,44,45,46,47,48], for review, see [30,31]):

*K*_d_[d(pN)*_n_*] = *K*_d_[(P_i_)] × (*E*)^1−*n*^ × (*h*_C_)^−*c*^ × (*h*_T_)^−*t*^ × (*h*_G_)^−*g*^ × (*h*_A_)^−*a*^ (1)

where *K*_d_ [(Pi)]—*K*_d_ for the minimal *ortho* phosphate ligand. The *h*_C_, *h*_T_, *h*_G_, and *h*_A_ values correspond to hydrophobic factors *h*_N_, reflecting an increase in the efficiency of the enzyme interaction due to the introduction of one of the bases (C, T, G, or A) into the d(pN)_n_ composition, the number of which in this ligand is *c*, *t*, *g*, and *a*, respectively. The relative values of the *h*_N_ factors very well correlate with the relative hydrophobicity of C, T, C, and A bases estimated using the isocratic reversed-phase chromatography [30]. The electrostatic factor *E* reflects an increase in the enzyme’s affinity due to the interaction of the enzyme or protein with one internucleoside phosphate group [30,31,32,33,34,35,36,37,38,39,40,41,42,43,44,45,46,47,48]. This equation describes the interaction of any ssDNA with any sequence-independent, as well as non-specific DNA with any of the investigated sequence-dependent enzymes. When passing from one to another enzyme or protein and from single- to double-stranded DNA, only a strong change in the numerical values of *K*_d_[(Pi)] and a slight change in the factors *E* and *h*_N_ are usually observed.

It is very interesting how histones in chromatin recognize DNA. However, this can be understood only by a sequential study of the patterns of complexation of each of the five free histones (H1, H2A, H2B, H3, and H4) with DNA, followed by the analysis of the histones complex interaction with DNA. This study’s aim was to analyze the interaction of DNA with free H1 histones by the method of a stepwise increase of the ligand complexity (SILC).

## 2. Results 

### Affinity H1 Histone for Different Single-Stranded Oligonucleotides

**Figure 1:**
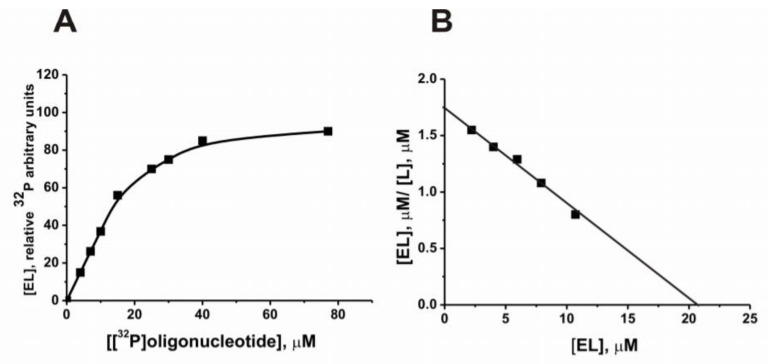
Dependence of the relative amount of [^32^P]ON (5′-[^32^P] TAGAAGATCAAA) bound to histone H1 on the concentration of the oligonucleotide (**A**). Eadie–Hofstee plot of the concentration of the complex ([EL], µM) on the ratio of the amount of the complex (µM) to the free ligand ([EL], µM/[L], µM) (**B**).

The binding of histone H1 with a 12-mer [^32^P]ON (5′-[^32^P]TAGAAGATCAAA-3′) was analyzed using the method of protein–ON complex delaying on nitrocellulose membrane filters [53]. Dependence of the relative amount of [^32^P]oligonucleotide bound to histone H1 on the concentration of the oligonucleotide is given in Figure 1A. To estimate the *K*_d_ value (12.0 ± 1.5 µM), such data were presented in Eadie–Hofstee coordinates (Figure 1B) according to [53]. To assess the affinity of H1 histone to oligonucleotides (ONs) of different structures and lengths, an inhibitory assay of the histone complex formation with [^32^P]ON was used.

It was previously shown that various dNMPs are minimal ligands of all DNA-binding enzymes and proteins ([30,31,32,33,34,35,36,37,38,39,40,41,42,43,44,45,46,47,48], for review, see [30,31]). The Gibbs free energy (∆G°) for a complex of ligand and protein is usually equal to the sum of the ∆G° values for all individual contacts:

∆G°_sum_ (corresponding to all *n* contacts) = ∆G°_1_ + ∆G°_2_ +… + ∆G°_n_ (2)

where all ∆G° correspond to all individual ligand contacts; ∆G° for each contact = −RT × ln*K*_d_. The total *K*_d_ value for the formation of the enzyme (protein)—ligand complex is the product of the *K*_d_ values for all individual contacts:

*K*_d_ = *K*_d_(1) × *K*_d_(2) × …… *K*_d_(n) (3)

**Figure 2:**
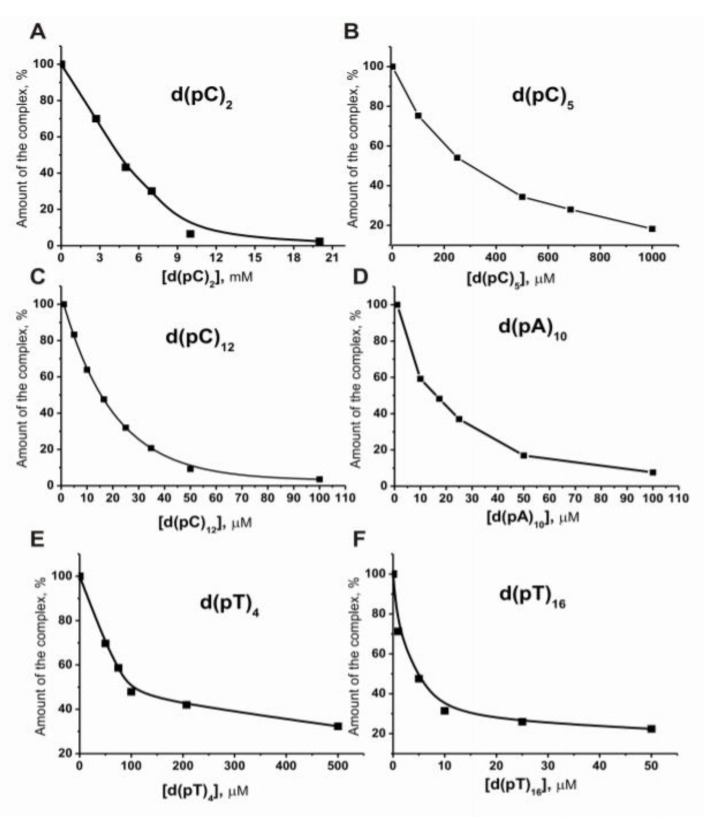
Dependence of the relative amount of [^32^P]ON (5′-[^32^P] TAGAAGATCAAA) bound to histone H1 (%) on the concentration of different single-stranded ON-inhibitors: d(pC)_2_ (**A**), d(pC)_5_ (**B**), d(pC)_12_ (**C**), d(pA)_10_ (**D**), d(pT)_4_ (**E**), d(pT)_16_ (**F**).

Using inhibitory analysis, the *I*_50_ values were first estimated for many single-stranded (ss) oligonucleotides: d(pC)_n_, d(pT)_n_, and d(pA)_n_. Figure 2 shows some typical examples of *I*_50_ values determination. When [^32^P]ON is used at a concentration equal to its dissociation constant (*K*_d_), the *I*_50_ values for competitive ligands are equal to their *K*_d_ values [53]. Using these *I*_50_ values, the *K*_d_ values were estimated for many oligonucleotides (ONs), which are summarized in Table 1.

As shown earlier, various dNMPs have an increased affinity specifically for active centers or specific sites of various enzymes and proteins (10^−5^−10^−1^ M) ([30,31,32,33,34,35,36,37,38,39,40,41,42,43,44,45,46,47,48], for review, see [30,31]). The remaining nucleotide units (7–20 depending on protein) of extended DNA usually interact with DNA-binding sites of all investigated enzymes and proteins [30,31,32,33,34,35,36,37,38,39,40,41,42,43,44,45,46,47,48] in an additive manner, demonstrating low affinity (the maximum affinity is characterized by *K*_d_ = 0.3–0.5 M [30,31]. As seen from Table 1, the minimal ligands of H1, as in the case of other DNA-dependent enzymes and proteins ([30,31,32,33,34,35,36,37,38,39,40,41,42,43,44,45,46,47,48], for review, see [30,31]), are also various dNMPs. The affinity of dCMP (1.0 × 10^−2^ M), dTMP (1.3 × 10^−2^ M) and dAMP (1.6 × 10^−2^ M) for H1 is comparable. 

For all studied enzymes, the dependences of −Lg*K*_d_ on the number of nucleotide units were linear at *n* ≥ 7–20 (depending on the enzyme molecular mass; 30–120 kDa), which indicated the additivity of Gibbs free energies characterizing their interaction with individual nucleotide units of d(pN)_n_ ([30,31,32,33,34,35,36,37,38,39,40,41,42,43,44,45,46,47,48,49,50,51,52], for review, see [30,31]). The data of Table 1 presented data on the *K*_d_ values for different single-stranded d(pN)_n_ homo-oligonucleotides and several hetero-ONs. One can see that *K*_d_ values for 12-rmer d(TAGAAGATCAAA) and other three 10-mer hetero-ONs are very comparable with those of homo-oligonucleotides of the same length. All data of Table 1 were presented in the form of logarithmic dependencies of *K*_d_ values on the number of mononucleotide units (Figure 2). 

The Equation (1) (see above) usually describes the interaction of any ss homo- and hetero-ONs with all sequence-independent enzymes, as well as unspecific oligonucleotides with sequence-dependent enzymes ([30,31,32,33,34,35,36,37,38,39,40,41,42,43,44,45,46,47,48,49,50,51,52], for review, see [30,31]). Only a numerical hydrophobic (*h*_N_) and electrostatic (*E*) factors are usually different. For example, in the case of DNA polymerases, topoisomerase, and apurinic apyrimidinic endonuclease (AP-EN), the *E* factors are equal to 1.52; 1.62, and 1.45, respectively; The *E* factor is usually higher than the *h*_N_ factor [30,31]. For example, the *h*_N_ factor for AP-EN is characterized by very low values (1.03–1.12) [46]. Moreover, Fpg protein [43,44] and *EcoRI* endonuclease [38] do not interact with DNA bases, and in the case of these enzymes, *h*_N_ = 1. 

**Figure 3:**
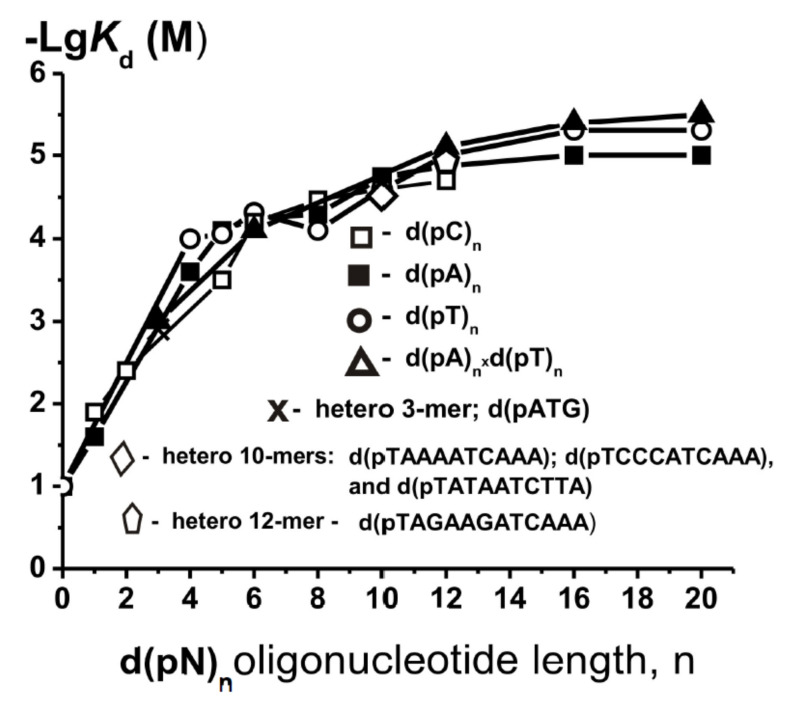
Dependences of negative logarithm values of *K*_d_ values on the number of nucleotide units (*n*) in the composition of some single- and double-stranded d(pN)_n_.

As seen from Figure 3, the dependences—Lg*K*_d_ on the number of nucleotide links (*n*) for all d(pN)_n_ practically coincide. This indicates that H1 histone does not form significant contacts with the bases of all ONs (factor *h*_N_ ≈ 1) and interacts mainly only with the sugar-phosphate backbones of these d(pN)_n_. In addition, it was previously shown that all enzymes and proteins mainly form weak contacts with internucleoside phosphate groups, but not with sugar residues of DNAs ([30,31,32,33,34,35,36,37,38,39,40,41,42,43,44,45,46,47,48,49,50,51,52], for review, see [30,31]).

When d(pN)_n_ is lengthened by one nucleotide unit to *n* = 7–20, a monotonic increase in affinity is usually observed, depending on the enzyme and DNA bases, by a factor of 1.2–2.6 [30,31]. Enzymes with molecular masses (MMs) of 30–40 kDa usually "cover" only 6–10 DNA nucleotide links. H1 has a molecular mass 23 kDa. Figure 3 demonstrates that the affinity of H1 for d(pN)_n_ effectively increases only up to *n* = 6, which is consistent with a relatively low molecular mass (MM) of this protein. 

From the slopes of the linear parts of the Lg-dependences, the values of the factors *f*, characterizing the increase in the affinity of proteins for different d(pN)_n_ with an increase in their length by one nucleotide unit can be calculated. The affinity of previously studied proteins and enzymes for ss ONs upon their lengthening by one unit increased by 1.2–2.7 times (factor *f*), Such factor for different proteins correspond to the values of *K*_d_ = 1/*f* = 0.37–0.83 M and the values of Gibbs free energy, equal to −0.12–−0.45 kcal/mol, reflecting the efficiency of the interaction of the enzyme with one unit of ssDNA.

Hydrogen and ionic bonds usually refer to strong interactions. The ∆G° values characterizing the formation of strong electrostatic contacts (from −1 to −2 kcal/mol), as well as hydrogen bonds (from −1 to −5 kcal/mol), can be relatively large. For simplicity of presentation, all types of weak interactions characterized by small values of Gibbs free energies (from −0.01 to −0.5 kcal/mol) may be formally considered as weak interactions. The ∆G° values for such contacts are significantly lower than those upon the formation of strong electrostatic contacts or hydrogen bonds between enzymes and ligands and are comparable with the values corresponding to weaker hydrophobic, ion-dipole, and dipole-dipole interactions [53].

From the data in Figure 3, it is possible to estimate the average value of factor *f* for H1 histone with the elongation of three types of d(pN)_n_ from 1 to 6 nucleotide links; *f* = 3.0 ± 0.2, and the *K*_d_ value characterizing the affinity of one nucleotide link at *n* = 2–6 is approximately 0.33 M. This affinity refers to weak interactions. Considering the literature data, this *K*_d_ values most likely (as in the case of other proteins and enzymes) characterize the efficiency of the interaction of H1 histone with one internucleoside phosphate group of ss oligonucleotides.

As shown earlier, the addition of a second DNA strand complementary to the first one can have different effects on the affinity of the duplex compared to ssDNA. The second DNA strand markedly increases the affinity of the duplex compared to ssDNA in the case of DNA polymerases [34,35,36,37], AP-EN [46], Fpg protein [43,44], topoisomerase I [40,41], HIV-1 integrase [39], and lactoferrin [49], but does not make a perceptible contribution to the affinity of the duplex for *EcoRI* endonuclease [38], uracil-DNA glycosylase [42], and human serum albumin [51]. While the first strand provides 5–9 orders of the total affinity of dsDNA, the contribution of the second strand usually varies mostly from 1.5 to 10 times and does not exceed 1–1.5 orders of magnitude ([30,31,32,33,34,35,36,37,38,39,40,41,42,43,44,45,46,47,48], for review, see [30,31]). This is consistent with the data of X-ray structural analysis that enzymes and proteins usually form the most important contacts only with one of the duplex chains ([30,31,32,33,34,35,36,37,38,39,40,41,42,43,44,45,46,47,48], for review, see [30,31]). In addition, ds d(pN)_n_ showed a significantly lower affinity for lactalbumin comparing with ss oligonucleotides [50].

**Figure 4:**
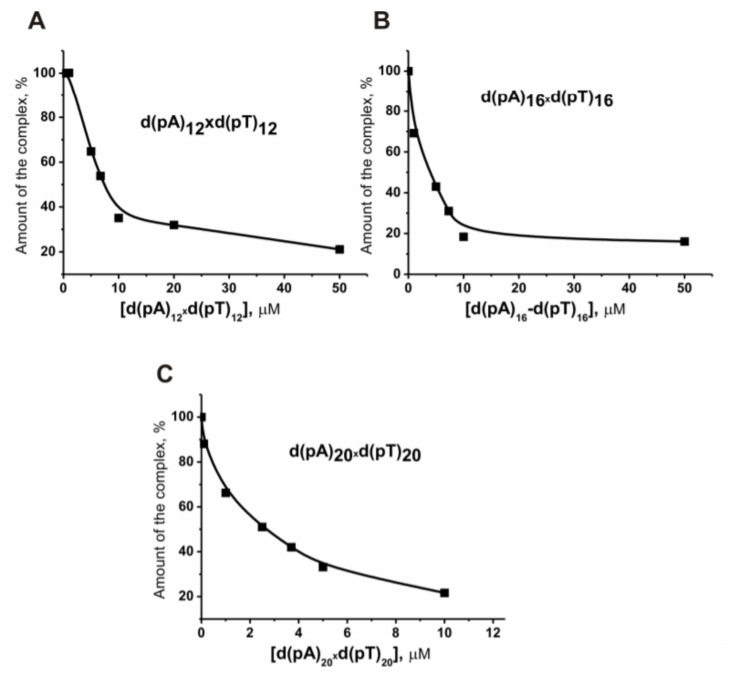
Dependence of the relative amount of [^32^P]ON (5′-[^32^P] TAGAAGATCAAA) bound to histone H1 (%) on the concentration of different double-stranded ON-inhibitors: d(pA)_12_ × d(pT)_12_ (**A**), d(pA)_16_ × d(pT)_16_ (**B**), and d(pA)_20_ × d(pT)_20_ (**C**).

We have estimated the affinity (*K*_d_ values) of mixtures of d(pT)_n_ and d(pA)_n_ containing 3, 6, 12, 16, and 20 nucleotide units (for example, Figure 4 and Table 1). The *K*_d_ values demonstrated by mixtures of complementary d(pN)_3_ and d(pN)_6_, as well as preformed duplexes d(pT)_n_ × d(pA)_n_ containing 12–20 nucleotide units, were comparable to the *K*_d_ values for corresponding ss d(pT)_n_ and d(pA)_n_. 

In addition, we evaluated the affinity of four ss 20-mer hetero-oligonucleotides (Table 1). All four oligonucleotides showed approximately the same affinity [(3.7–4.1) × 10^−6^ M) comparable to that for d(pC)_20_ and d(pT)_20_ [(4.4–4.6) × 10^−6^ M]. From these four pairwise complementary oligonucleotides, two preformed duplexes were obtained. These two duplexes showed approximately the same affinity [(2.7–2.9) × 10^−6^ M] for H1 histone (Table 1). Wherein, their affinity for histone H1 was only 1.3–1.5-fold higher than for ss oligonucleotides included in their composition. Interestingly, the affinity of both 20-mer hetero-duplexes was comparable to that for d(pA)_20_ × d(pT)_20_ (3.0 × 10^−6^ M), demonstrating 1.5–3.3-fold difference in duplex affinity compared to ss d(pA)_20_ and d(pT)_20_ oligonucleotides. This indicates that the second strand of ds DNA may have little effect on the affinity of its first strand. This could be because, like *EcoRI* endonuclease [38], uracil-DNA glycosylase [42], and human serum albumin [51], free H1 histone predominantly forms contacts with only one of the duplex strands.

## 3. Discussion

It is known that structural elements of some low-molecular-weight ligands and substrates form very strong bonds with enzymes, 10^−5^–10^−6^ M [30,31,53]. However, a very high affinity of enzymes and proteins for specific sequences or nucleotide units of extended DNAs can be dangerous for living organisms. For example, repair enzymes show a high affinity for extended DNA, 10^−7^–10^−9^ M. If such a high affinity is provided by the interaction of enzymes with only specific modified nucleotide units, after their removal from DNA, enzymes could be significantly inhibited by free mononucleotides. It is known that the rate of action of many enzymes is very high. Many studies of enzymes have shown that to ensure a high reaction rate, enzymes slide along DNA when searching for specific sequences and/or structural elements (specific sequences, single-stranded DNA fragments, modified nucleotides, breaks, etc.) ([30,31,32,33,34,35,36,37,38,39,40,41,42,43,44,45,46,47,48], for review, see [30,31]). Such sliding can only be achieved if the enzymes can bind to the DNA of any sequence with sufficiently high affinity. Therefore, significant differences in the enzymes’ affinity for unspecific and specific DNA can lead to a strong suppression in the sliding speed and, as a consequence, to the reaction rate decrease.

To reveal the most important factors are providing specificity, first, we analyzed many enzymes: DNA repair, DNA replication, topoisomerization, integration and recombination using different physiochemical approaches, including the SILC method ([30,31,32,33,34,35,36,37,38,39,40,41,42,43,44,45,46,47,48], for review, see [30,31]). It was demonstrated that a high affinity of long DNA is provided by the formation of many weak additive hydrophobic and/or van der Waals interactions of all studied enzymes with all nucleotide links covered by enzyme globules. Depending on the enzyme, the sum of weak non-specific additive contacts provides 5–9 orders of the enzyme affinity for specific and unspecific DNAs ([30,31,32,33,34,35,36,37,38,39,40,41,42,43,44,45,46,47,48], for review, see [30,31]). In contrast to enzymes interacting with small ligands, all interactions of enzymes with specific structural elements of long DNA are usually weak, and their efficiencies are comparable with weak additive non-specific contacts. All specific interactions between DNA and enzymes usually provide approximately only one [30,31] and rarely about two orders of the affinity [38,45]. According to X-ray data, after DNA binding with different enzymes, there is a stage of very specific DNA and enzyme conformation adjustment (for review, see [30,31]). Depending on the enzyme, there may be deformation of DNA backbone, its stretching or compression, partial or complete DNA melting, bending or kinking, eversion of nucleotides from the DNA helix, etc. ([30,31,32,33,34,35,36,37,38,39,40,41,42,43,44,45,46,47,48], for review, see [30,31]). Such changes in DNA are very specific for each individual enzyme. Enzyme-dependent specific changes in the conformation of DNAs are required for effective adjustment of enzyme and DNA reacting orbitals with accuracy about 10–15 degrees ([30,31,32,33,34,35,36,37,38,39,40,41,42,43,44,45,46,47,48], for review, see [30,31,53]), which is possible only for specific DNA. The transition from unspecific to specific DNAs usually leads to the rise in the reaction rate (*k*_cat_) by 5–8 orders of magnitude. Taken together, the stages of enzyme-dependent adjustment of DNA conformation and directly catalysis provide the high specificity of enzymes’ actions ([30,31,32,33,34,35,36,37,38,39,40,41,42,43,44,45,46,47,48], for review, see [30,31]). 

Unlike enzymes, many proteins recognizing DNA lack catalytic activity. Therefore, it was interesting to understand whether there is any difference in the patterns of recognition of specific and non-specific DNA by enzymes and such proteins. We first analyzed DNA recognition by human lactoferrin [49], lactalbumin [50], serum albumin [51], and IgGs against DNA [52] using the SILC approach. It turned out that all these proteins, including antibodies, recognize DNA in accordance with the general laws described above. The formation of additional contacts between lactoferrin and its DNA specific sequence (like for other sequence-specific enzymes [30,31,32,33,34,35,36,37,38,39,40,41,42,43,44,45,46]) led to the increase of the DNA affinity in comparison with unspecific ones by approximately one order of magnitude. 

Linker histone H1 is one of the most abundant chromatin components [54,55,56]. H1 binds to DNA entering and exiting the nucleosome particle nucleus and plays an important role in the creation and maintenance of higher-order chromatin structures [54,55,56]. H1 has a profound effect on chromosome architecture. The linker histone binds to the nucleosome to form the next structural unit of chromatin, the chromatosome [54]. H1 also helps bind DNA and histone-modifying enzymes to chromatin [55]. H1 interacts directly with Suv39h1, Suv39h2, and SETDB1, histone methyltransferases responsible for trimethylation of H3K9 chromatin in these regions and stimulates their activity towards chromatin in vitro [56]. The interaction of the human linker histone H1° with short oligonucleotides has been characterized [57]. The ability of histone to promote the exchange of DNA strands in this system has been demonstrated. The reaction is reversible at saturating amounts of H1 corresponding to the complete binding of oligonucleotide substrates with histone. It has been shown that the linker histone H1 performs its numerous biological functions through independent, biochemically different activities of its individual structural domains [55].

However, the patterns of DNA recognition by a complex of histones of the cell nucleus are of particular interest. The analysis of the peculiarities of the interaction of DNA with the complex of all five nuclear histones seems to be rather complicated. First, it was important to understand how each of the five histones can recognize DNA, and only then, through synthesis and analysis, it will be possible to understand the principles of the organization of the DNA complex with five histones. 

In this work, we have analyzed the patterns of DNA recognition by free H1 histone. There is data on H1 binding to DNA, nucleosomes, or chromatin [54,55,56]. It was shown that H1 histone bound to nucleosomes with DNA is mostly electrostatic [27,28]. However, in these publications, there is no more detailed quantitative data on how free H1° recognizes DNA, including its possible interactions with nucleotide bases or preferred DNA sequences as well as the number of nucleotide DNA units covered by the protein.

It turned out that the patterns of DNA recognition by histone H1 do not differ from those for previously studied enzymes and proteins. As in the case of other enzymes and proteins ([30,31,32,33,34,35,36,37,38,39,40,41,42,43,44,45,46,47,48,49,50,51,52], for review, see [30,31]), the minimal ligands of H1 histone are deoxymononucleotides (Table 1). Interestingly, the affinity of dAMP, dTMP, and dCMP for this histone is comparable.

The dependences—Lg*K*_d_ on the number of nucleotide links (*n*) for d(pA)_n_, d(pT)_n_, d(pC)_n_, and hetero-d(pN)_n_ almost coincide. It means that H1 histone does not interact with the DNA bases (factor *h*_N_ = 1). H1 interacts only with the sugar-phosphate backbone of ss DNA; electrostatic factor *E* = 3.0 ± 0.2; the *K*_d_ value characterizing the affinity of one internucleoside phosphate group of ssDNA at *n* = 2–6 is approximately 0.33 M.

Interestingly, all the studied enzymes and proteins usually have one specific subsite in an extended “channel” for DNA recognition with an increased affinity of only one nucleotide unit of DNA ([30,31,32,33,34,35,36,37,38,39,40,41,42,43,44,45,46,47,48], for review, see [30,31]). This is also true for H1 histone. The affinity of one subsite to dAMP, dTMP, and dCMP (*K*_d_ = (1.0 − 1.6) × 10^−2^ M) is about 20–33-fold higher than the other 2–6 sites (*K*_d_ = 0.33 M).

The *K*_d_ values for d(pT)_12–20_ × d(pA)_12–20_ duplexes are comparable with the *K*_d_ values for corresponding ss d(pT)_n_ and d(pA)_n_. *EcoRI* endonuclease [38] and uracil-DNA glycosylase [42] completely melt specific dsDNAs for their recognition. One cannot exclude that H1 histone also forms contacts with only one of the duplex strands.

Enzymes and proteins with MMs of 30–40 kDa, usually “cover” only 6–10 nucleotide links of DNA. H1 has a molecular mass 23 kDa. Therefore, one would expect that histone H1 can form contacts with only 5–6 nucleotide units of DNA. As seen from Figure 3, the affinity of H1 for d(pN)_n_ effectively increases only up to *n* = 6, which is consistent with a relatively low MM of this protein. At the same time, the Lg*K*_d_ values for the previously studied enzymes usually remain constant after reaching a plateau after *n* = 7–20 ([30,31,32,33,34,35,36,37,38,39,40,41,42,43,44,45,46,47,48,49,50,51,52], for review, see [30,31]). In the case of H1 at *n* > 6, a noticeable increase in the ONs affinity still occurs (Figure 3). It may be a consequence of the fact that under the conditions used (0.3 mg/mL H1), part of the protein molecules may be in the dimeric state, as well as in other oligomeric forms [9,10]. This can lead to a slight increase in the affinity for d(pN)_n_ at *n* > 6 due to the interaction of oligonucleotides simultaneously with several globules of oligomeric forms of H1 histone. A similar situation was observed earlier for lactalbumin (14.1 kDa), forming dimer and tetramer complexes of the protein [50].

## 4. Materials and Methods 

### 4.1. Chemicals 

Most chemicals, including Tris (No. 252859), MgCl_2_ (No. 449172), EDTA (No. E4884) used were provided by Sigma-Aldrich (St. Louis, MO, USA). Homogeneous human recombinant H1° histone (M2501S) was from BioLabs (New York, USA). All ONs were prepared from commercially available phosphoramidites (Glen Research, Sterling, VA) using ASM-800 synthesizers (BIOSSET, Novosibirsk, Russia). The sequences of ONs used are given in several Table 1. All oligonucleotides were homogeneous as judged by reversed-phase and ion-exchange chromatography and electrophoresis in 20% polyacrylamide gel. Phosphorylation of ONs was carried out according to [58] by transferring the terminal phosphate from [γ-^32^P]-ATP to the 5′ position of the oligonucleotide using polynucleotide kinase of the phage T4 from Biosan (Novosibirsk, Russia).

### 4.2. Analysis of the Binding of Histones to DNA on Membrane Filters

The binding of histone H1 to a hetero-ON (5′-[^32^P] TAGAAGATCAAA-3′) was assessed by the method of protein–ON complex delaying on nitrocellulose membrane filters Synpor No. 6 (Czech Republic). These filters effectively trap proteins with molecular masses ≥10 kDa. The reaction mixture (10 μL) contained 20 mM Tris-HCl (pH 7.5), 5 mM MgCl_2_, 1 mM EDTA, 0.3 mg/mL histone H1, and hetero-ON at various concentrations: 10^−8^–10^−3^ M. The reaction mixtures were incubated during 1 h at 20 °C, and 3 μL of them were applied to the filters; the solution was evacuated using a vacuum pump, and the filters were washed with 3.2 mL of buffer containing 20 mM Tris-HCl (pH 7.5), 5 mM MgCl_2_, 1 mM EDTA. The filters were dried and visualized by radioluminescence scanning on an Image Screen K using a Typhoon FLA 9500 system. Quantitative processing of the results was carried out using the ImageQuant v5.2 software (UK, River Tyne). To determine the *K*_d_ value of the H1 complex with hetero-ON, the dependence of the relative amount of the radioactive label on the concentration of the oligonucleotide was used. The dissociation value constant (*K*_d_ = 12.0 ± 1.3 μM) was found using Eadie–Hofstee plot of [EL] against [EL]/[L] (Figure 1B) according to [53].

### 4.3. Preparation of Oligonucleotides Duplexes

The duplexes of ONs were prepared by standard method. The formation of duplexes and determination of their melting points (T_m_) were carried out under the same conditions; 50 mM cacodylic buffer pH 7.4, 50 mM NaCl. Mixtures of complementary ONs in equal concentrations were heated for 1 min at 90 °C, followed by slow cooling to 20 °C. Evaluation of melting points was performed using Cary 300 Bio UV-Vis Spectrophotometer (Varian, Australia). The next T_m_ values were found: d(pA)_16_ × d(pT)_16_ (28 °C), d(pA)_20_ × d(pT)_20_ (38 °C), duplex d(CAGACGATCAGCGACGCGTC)×complementary ODNcom1 (64 °C), duplex d(AGTGCCTGACCGTCGTCGAC)×complementary ODNcom2 (66 °C). In the case of all four duplexes, only one melting point was found. This indicated that they were all correctly formed. The reaction mixtures containing 20 mM Tris-HCl (pH 7.5), 5 mM MgCl_2_, 1 mM EDTA, the duplexes, and H1 histone were incubated at 20 °C, which is significantly lower than the T_m_ of duplexes.

### 4.4. Determination of H1 Histone Affinity for Different Oligonucleotides

Assessment of the H1 affinity for ONs of various structures and lengths was carried out using an inhibitory assay. The reaction mixture (10 μL) contained all the same standard components as in the case of the assessment of the H1 affinity for [^32^P]ON, as well as 0.3 mg/ml of histone H1 and 12.0 μM 5′-[^32^P] TAGAAGATCAAA (at a concentration equal to the *K*_d_ value for the complex of this oligonucleotide with H1). ONs of various structures and lengths were also added to the reaction mixtures at various concentrations from 10^−7^ to 10^−3^ M. The reaction mixtures were incubated, applied to filters, and the rest of the operations were carried out as described above. The quantitative processing of the intensity of the spots was carried out using the ImageQuant v5.2 software. To determine the dissociation constant of the H1 complex with different ONs, the dependence of the relative amount of the radioactive label on the concentration of ON inhibitors of complex formation was used. From these dependencies, the *I*_50_ value was estimated (ON concentrations at which the amount of the complex with the radioactive ligand decreases by 50%). When [^32^P]ON is used at a concentration equal to its dissociation constant (*K*_d_), the *I*_50_ values for competitive ligands are equal to their *K*_d_ values [53].

## 5. Conclusions 

In this work, we first have performed quantitative analysis of the recognition by free H1° histone of various single- and double-stranded oligonucleotides depending on their sequences and length. It was shown that H1 forms weak electrostatic additive contacts with six internucleoside phosphate groups of single-stranded and only one chain of double-stranded DNAs, but not with their bases.

## Figures and Tables

**Table 1:** *K*_d_ values characterizing the affinity of various oligonucleotides for H1 histone.

Ligand	*K*_d_, M *	Ligand	*K*_d_, M *	Ligand	*K*_d_, M
dAMP	1.6 × 10^−2^	dCMP	1.0 × 10^−2^	dTMP	1.3 × 10^−2^
d(pA)_4_	2.4 × 10^−4^	d(pC)_2_	4.4 × 10^−3^	d(pT)_4_	9.6 × 10^−5^
d(pA)_5_	7.0 × 10^−5^	d(pC)_5_	3.0 × 10^−4^	d(pT)_5_	7.5 × 10^−5^
d(pA)_6_	5.7 × 10^−5^	d(pC)_6_	6.1 × 10^−5^	d(pT)_6_	4.8 × 10^−5^
d(pA)_8_	4.8 × 10^−5^	d(pC)_8_	3.4 × 10^−5^	d(pT)_8_	7.6 × 10^−5^
d(pA)_10_	2.8 × 10^−5^	d(pC)_10_	2.5 × 10^−5^	d(pT)_10_	2.5 × 10^−5^
d(pA)_12_	1.4 × 10^−5^	d(pC)_12_	2.0 × 10^−5^	d(pT)_12_	1.0 × 10^−5^
d(pA)_16_	1.0 × 10^−5^	_–_	-	d(pT)_16_	5.0 × 10^−6^
d(pA)_20_	1.0 × 10^−5^	d(pC)_20_	4.4 × 10^−6^	d(pT)_20_	4.6 × 10^−6^
d(pATG)	1.5 × 10^−3^	d(TAAAATCAAA)	3.0 × 10^−5^	d(TCCCATCAAA)	3.0 × 10^−5^
d(TATAATCTTA)	2.8 × 10^−5^	d(TAGAAGATCAAA)	1.2 × 10^−5^		
20-mer ODN1 d(CAGACGATCAGCGACGCGTC)	4.0 × 10^−6^	20-mer ODN2 ** d(AGTGCCTGACCGTCGTCGAC)	3.8 × 10^−6^	20-mer ODNcom1 ** d(GTCTGCTAGTCGCTGCGCAG)	3.7 × 10^−6^
20-mer ODNcom2 d(TCACGGACTGGCAGCAGCTG)	4.1 × 10^−6^	-	-	-	-
Oligonucleotide Mixtures and Duplexes					
d(pA)_3_ × d(pT)_3_	1.0 × 10^−3^	d(pA)_6_ × d(pT)_6_	5.5 × 10^−5^	d(pA)_12_ × d(pT)_12_	7.5 × 10^−6^
d(pA)_16_ × d(pT)_16_	3.8 × 10^−6^	d(pA)_20_ × d(pT)_20_	3.0 × 10^−6^	-	-
20-mer duplex d(CAGACGATCAGCGACGCGTC) × ODNcom1 ***	2.7 × 10^−6^	20-mer duplex d(AGTGCCTGACCGTCGTCGAC) × ODNcom2 ***	2.9 × 10^−6^		

* The error in determining the values from the data of three experiments did not exceed 10–12%. ** 20-mer ODNcom1 and 20-mer ODNcom2 are oligonucleotides complementary to 20-mer ODN1 and 20-mer ODN2, respectively. *** Duplexes of ODN1 and ODN2 with corresponding complementary oligonucleotides.

## Abbreviations

AP-EN	human apurinic/apyrimidinic endonuclease
Fpg	E. coli formamidopyrimidine or 8-oxoguanine DNA glycosylase
I_50_	inhibitor concentration reducing complexation by 50%
dNMPs	5′-dexosimononucleotides
d(pN)n and ON	oligodeoxyribonucleotide
ss and ds	single- and double-stranded
SILC	stepwise increase in ligand complexity
topoisomerase I	human topoisomerase I
Pi	inorganic orthophosphate
kDa	kilo Daltons
dNMPs	nucleoside monophosphate
RA	relative activity
